# 4-Chloro-*N*-(2,6-dimethyl­phen­yl)benzene­sulfonamide

**DOI:** 10.1107/S160053681101717X

**Published:** 2011-05-14

**Authors:** K. Shakuntala, Sabine Foro, B. Thimme Gowda

**Affiliations:** a Department of Chemistry, Mangalore University, Mangalagangotri 574 199, Mangalore, India; b Institute of Materials Science, Darmstadt University of Technology, Petersenstrasse 23, D-64287 Darmstadt, Germany

## Abstract

In the title compound, C_14_H_14_ClNO_2_S, the amido H atom orients itself away from both the *ortho*-methyl groups in the adjacent aromatic ring. The mol­ecule is twisted at the S atom with an C—SO_2_—NH—C torsion angle of −69.9 (2)°. The two aromatic rings are tilted relative to each other by 31.9 (1)°. In the crystal, the mol­ecules are packed into zigzag chains along the *b* axis *via* inter­molecular N—H⋯O hydrogen bonds.

## Related literature

For hydrogen-bonding modes of sulfonamides, see; Adsmond & Grant (2001 ▶). For our study of the effect of substituents on the structures of *N*-(ar­yl)methane­sulfonamides, see: Gowda *et al.* (2007 ▶), on the structures of *N*-(ar­yl)aryl­sulfonamides, see: Gowda *et al.* (2008 ▶); Shakuntala *et al.* (2011 ▶) and on the oxidative strengths of *N*-chloro,*N*-aryl­sulfonamides, see: Gowda & Kumar (2003 ▶).
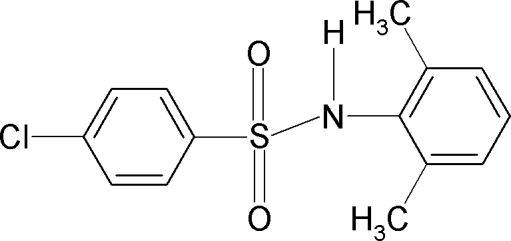


## Experimental

### 

#### Crystal data

C_14_H_14_ClNO_2_S
*M* *_r_* = 295.77
Orthorhombic, 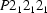

*a* = 7.3816 (4) Å
*b* = 10.2916 (7) Å
*c* = 18.312 (1) Å
*V* = 1391.13 (14) Å^3^
*Z* = 4
Mo *K*α radiation
μ = 0.42 mm^−1^
*T* = 293 K
0.40 × 0.28 × 0.24 mm

#### Data collection

Oxford Diffraction Xcalibur diffractometer with a Sapphire CCD detector
Absorption correction: multi-scan (*CrysAlis RED*; Oxford Diffraction, 2009 ▶) *T*_min_ = 0.850, *T*_max_ = 0.906
5356 measured reflections
2767 independent reflections
2255 reflections with *I* > 2σ(*I*)
*R*_int_ = 0.022

#### Refinement

*R*[*F* ^2^ > 2σ(*F* ^2^)] = 0.036
*wR*(*F* ^2^) = 0.079
*S* = 1.02
2767 reflections
177 parameters
1 restraint
H atoms treated by a mixture of independent and constrained refinement
Δρ_max_ = 0.19 e Å^−3^
Δρ_min_ = −0.25 e Å^−3^
Absolute structure: Flack (1983 ▶), 1113 Friedel pairs
Flack parameter: 0.43 (7)

### 

Data collection: *CrysAlis CCD* (Oxford Diffraction, 2009 ▶); cell refinement: *CrysAlis RED* (Oxford Diffraction, 2009 ▶); data reduction: *CrysAlis RED*; program(s) used to solve structure: *SHELXS97* (Sheldrick, 2008 ▶); program(s) used to refine structure: *SHELXL97* (Sheldrick, 2008 ▶); molecular graphics: *PLATON* (Spek, 2009 ▶); software used to prepare material for publication: *SHELXL97*.

## Supplementary Material

Crystal structure: contains datablocks I, global. DOI: 10.1107/S160053681101717X/bq2299sup1.cif

Structure factors: contains datablocks I. DOI: 10.1107/S160053681101717X/bq2299Isup2.hkl

Supplementary material file. DOI: 10.1107/S160053681101717X/bq2299Isup3.cml

Additional supplementary materials:  crystallographic information; 3D view; checkCIF report

## Figures and Tables

**Table 1:** Hydrogen-bond geometry (Å, °)

*D*—H⋯*A*	*D*—H	H⋯*A*	*D*⋯*A*	*D*—H⋯*A*
N1—H1*N*⋯O1^i^	0.82 (2)	2.28 (2)	3.083 (3)	166 (3)

Symmetry code: (i) 
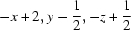
.

**Fig. 1.**
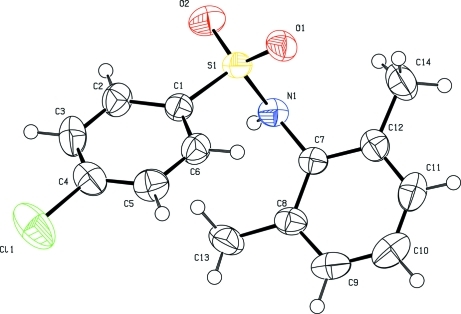
Molecular structure of (I), showing the atom labelling scheme and displacement ellipsoids are drawn at the 50% probability level.

**Fig. 2.**
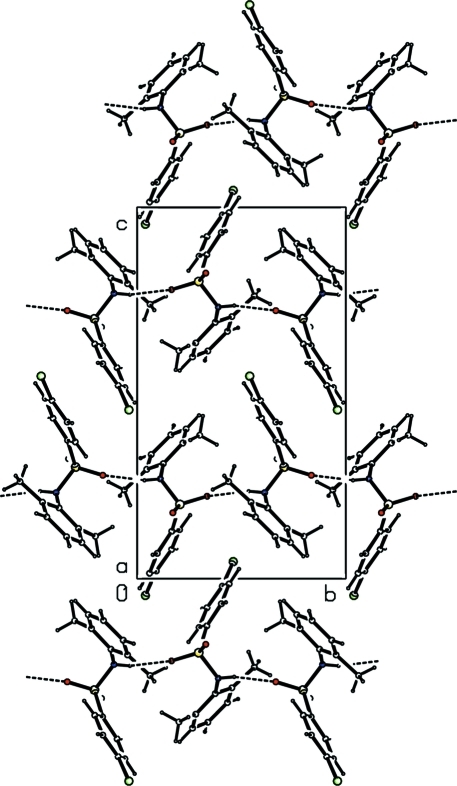
Molecular packing of (I) with hydrogen bonding shown as dashed lines.

## supplementary crystallographic information

### Comment

The sulfonamide moieties are the constituents of many biologically important compounds. The hydrogen bonding preferences of sulfonamides has been investigated (Adsmond & Grant, 2001). As a part of studying the substituent effects on the structures and other aspects of this class of compounds (Gowda *et al.*, 2003, 2007, 2008; Shakuntala *et al.*, 2011), in the present work, the crystal structure of 4-chloro-*N*-(2,6-dimethylphenyl)-benzenesulfonamide (I) has been determined (Fig.1). In the structure, the amido H atom orients itself away from both the *ortho*-methyl groups in the adjacent aromatic ring. The molecule is twisted at the S atom with the C—SO_2_—NH—C torsion angle of -69.9 (2)°, compared to the values of -53.8 (3)° (molecule 1) and -63.4 (3)° (molecule 2) in 4-chloro-*N*-(phenyl)-benzenesulfonamide (II) (Shakuntala *et al.*, 2011) and -78.7 (2)° in *N*-(2,6-dimethylphenyl)-benzenesulfonamide (III) (Gowda *et al.*, 2008)

The sulfonyl and anilino benzene rings in (I) are tilted relative to each other by 31.9 (1)°, compared to the values of 69.1 (1)° in molecule 1 and 82.6 (1)° in molecule 2 of (II), and 44.9 (1)° in (III).

The packing of molecules in (I) into zigzag chains through N—H···O(S) hydrogen bonding (Table 1) is shown in Fig.2.

### Experimental

The solution of chlorobenzene (10 ml) in chloroform (40 ml) was treated dropwise with chlorosulfonic acid (25 ml) at 0 ° C. After the initial evolution of hydrogen chloride subsided, the reaction mixture was brought to room temperature and poured into crushed ice in a beaker. The chloroform layer was separated, washed with cold water and allowed to evaporate slowly. The residual 4-chlorobenzenesulfonylchloride was treated with 2,6-dimethylaniline in the stoichiometric ratio and boiled for ten minutes. The reaction mixture was then cooled to room temperature and added to ice cold water (100 ml). The resultant 4-chloro-*N*-(2,6-dimethylphenyl)-benzenesulfonamide was filtered under suction and washed thoroughly with cold water. It was then recrystallized to constant melting point from dilute ethanol. The compound was characterized by recording its infrared and NMR spectra.

Prism like colorless single crystals used in X-ray diffraction studies were grown in ethanolic solution by slow evaporation at room temperature.

### Refinement

The H atom of the NH group was located in a difference map and later restrained to the distance N—H = 0.86 (2) Å. The other H atoms were positioned with idealized geometry using a riding model with the aromatic C—H = 0.93 Å and the methyl C—H = 0.96 Å. All H atoms were refined with isotropic displacement parameters (set to 1.2 times of the *U*_eq_ of the parent atom).

### Crystal data

**Table:**

C_14_H_14_ClNO_2_S	*F*(000) = 616
*M**_r_* = 295.77	*D*_x_ = 1.412 Mg m^−^^3^
Orthorhombic, *P*2_1_2_1_2_1_	Mo *K*α radiation, λ = 0.71073 Å
Hall symbol: P 2ac 2ab	Cell parameters from 1775 reflections
*a* = 7.3816 (4) Å	θ = 2.8–28.0°
*b* = 10.2916 (7) Å	µ = 0.42 mm^−^^1^
*c* = 18.312 (1) Å	*T* = 293 K
*V* = 1391.13 (14) Å^3^	Prism, colourless
*Z* = 4	0.40 × 0.28 × 0.24 mm

### Data collection

**Table:**

Oxford Diffraction Xcalibur diffractometer with a Sapphire CCD detector	2767 independent reflections
Radiation source: fine-focus sealed tube	2255 reflections with *I* > 2σ(*I*)
graphite	*R*_int_ = 0.022
Rotation method data acquisition using ω and φ scans	θ_max_ = 26.4°, θ_min_ = 3.0°
Absorption correction: multi-scan (*CrysAlis RED*; Oxford Diffraction, 2009)	*h* = −9→6
*T*_min_ = 0.850, *T*_max_ = 0.906	*k* = −12→10
5356 measured reflections	*l* = −22→17

### Refinement

**Table:**

Refinement on *F*^2^	Secondary atom site location: difference Fourier map
Least-squares matrix: full	Hydrogen site location: inferred from neighbouring sites
*R*[*F*^2^ > 2σ(*F*^2^)] = 0.036	H atoms treated by a mixture of independent and constrained refinement
*wR*(*F*^2^) = 0.079	*w* = 1/[σ^2^(*F*_o_^2^) + (0.0326*P*)^2^ + 0.3144*P*] where *P* = (*F*_o_^2^ + 2*F*_c_^2^)/3
*S* = 1.02	(Δ/σ)_max_ < 0.001
2767 reflections	Δρ_max_ = 0.19 e Å^−^^3^
177 parameters	Δρ_min_ = −0.25 e Å^−^^3^
1 restraint	Absolute structure: Flack (1983), 1113 Friedel pairs
Primary atom site location: structure-invariant direct methods	Flack parameter: 0.43 (7)

### Special details

**Table:**

Experimental. CrysAlis RED (Oxford Diffraction, 2009) Empirical absorption correction using spherical harmonics, implemented in SCALE3 ABSPACK scaling algorithm.
Geometry. All e.s.d.'s (except the e.s.d. in the dihedral angle between two l.s. planes) are estimated using the full covariance matrix. The cell e.s.d.'s are taken into account individually in the estimation of e.s.d.'s in distances, angles and torsion angles; correlations between e.s.d.'s in cell parameters are only used when they are defined by crystal symmetry. An approximate (isotropic) treatment of cell e.s.d.'s is used for estimating e.s.d.'s involving l.s. planes.
Refinement. Refinement of *F*^2^ against ALL reflections. The weighted *R*-factor *wR* and goodness of fit *S* are based on *F*^2^, conventional *R*-factors *R* are based on *F*, with *F* set to zero for negative *F*^2^. The threshold expression of *F*^2^ > σ(*F*^2^) is used only for calculating *R*-factors(gt) *etc*. and is not relevant to the choice of reflections for refinement. *R*-factors based on *F*^2^ are statistically about twice as large as those based on *F*, and *R*- factors based on ALL data will be even larger.

### Fractional atomic coordinates and isotropic or equivalent isotropic displacement parameters (Å^2^)

**Table:**

	*x*	*y*	*z*	*U*_iso_*/*U*_eq_	
Cl1	0.42735 (14)	0.03948 (8)	−0.04544 (5)	0.0851 (3)	
S1	0.96404 (8)	0.20494 (6)	0.19969 (4)	0.04077 (16)	
O1	0.9231 (3)	0.33512 (15)	0.22176 (9)	0.0502 (5)	
O2	1.1425 (2)	0.1712 (2)	0.17729 (11)	0.0606 (6)	
N1	0.9167 (3)	0.11125 (19)	0.26858 (12)	0.0389 (5)	
H1N	0.950 (3)	0.0356 (17)	0.2645 (14)	0.047*	
C1	0.8156 (3)	0.1660 (2)	0.12743 (13)	0.0375 (6)	
C2	0.8780 (4)	0.0962 (2)	0.06798 (14)	0.0479 (7)	
H2	1.0000	0.0745	0.0642	0.057*	
C3	0.7573 (4)	0.0591 (3)	0.01437 (15)	0.0568 (8)	
H3	0.7974	0.0119	−0.0258	0.068*	
C4	0.5788 (4)	0.0917 (3)	0.02047 (14)	0.0513 (7)	
C5	0.5165 (4)	0.1648 (3)	0.07828 (15)	0.0524 (7)	
H5	0.3949	0.1880	0.0811	0.063*	
C6	0.6357 (3)	0.2032 (3)	0.13178 (14)	0.0450 (6)	
H6	0.5958	0.2539	0.1706	0.054*	
C7	0.7516 (3)	0.1254 (2)	0.30961 (13)	0.0357 (5)	
C8	0.6035 (3)	0.0460 (2)	0.29430 (14)	0.0424 (6)	
C9	0.4451 (4)	0.0670 (3)	0.33397 (15)	0.0560 (7)	
H9	0.3433	0.0166	0.3244	0.067*	
C10	0.4376 (4)	0.1614 (3)	0.38707 (17)	0.0620 (8)	
H10	0.3302	0.1752	0.4124	0.074*	
C11	0.5853 (4)	0.2345 (3)	0.40283 (14)	0.0555 (8)	
H11	0.5782	0.2965	0.4397	0.067*	
C12	0.7464 (4)	0.2189 (3)	0.36519 (13)	0.0433 (6)	
C13	0.6103 (4)	−0.0616 (3)	0.23877 (15)	0.0573 (7)	
H13A	0.6806	−0.0340	0.1975	0.069*	
H13B	0.4896	−0.0823	0.2232	0.069*	
H13C	0.6652	−0.1371	0.2602	0.069*	
C14	0.9069 (4)	0.2998 (3)	0.38620 (16)	0.0642 (8)	
H14A	1.0162	0.2550	0.3730	0.077*	
H14B	0.9055	0.3145	0.4380	0.077*	
H14C	0.9017	0.3816	0.3611	0.077*	

### Atomic displacement parameters (Å^2^)

**Table:**

	*U*^11^	*U*^22^	*U*^33^	*U*^12^	*U*^13^	*U*^23^
Cl1	0.1151 (8)	0.0671 (5)	0.0730 (6)	−0.0104 (5)	−0.0493 (5)	0.0049 (4)
S1	0.0373 (3)	0.0394 (3)	0.0457 (3)	−0.0063 (3)	0.0044 (3)	−0.0015 (3)
O1	0.0686 (12)	0.0340 (9)	0.0480 (10)	−0.0116 (9)	0.0022 (9)	0.0000 (8)
O2	0.0365 (10)	0.0769 (15)	0.0684 (13)	−0.0049 (9)	0.0078 (8)	−0.0039 (11)
N1	0.0376 (12)	0.0320 (11)	0.0470 (12)	0.0027 (9)	0.0007 (9)	0.0018 (10)
C1	0.0416 (14)	0.0321 (13)	0.0389 (13)	−0.0015 (11)	0.0030 (11)	0.0013 (11)
C2	0.0529 (16)	0.0410 (15)	0.0498 (16)	0.0081 (12)	0.0069 (13)	−0.0040 (13)
C3	0.084 (2)	0.0459 (17)	0.0407 (15)	0.0074 (17)	0.0016 (15)	−0.0071 (13)
C4	0.067 (2)	0.0398 (15)	0.0468 (16)	−0.0057 (14)	−0.0123 (14)	0.0078 (13)
C5	0.0452 (15)	0.0573 (17)	0.0548 (16)	0.0013 (13)	−0.0048 (13)	0.0073 (14)
C6	0.0449 (15)	0.0453 (15)	0.0446 (14)	0.0040 (13)	0.0062 (12)	−0.0026 (13)
C7	0.0363 (12)	0.0346 (13)	0.0360 (13)	−0.0003 (10)	−0.0034 (10)	0.0064 (11)
C8	0.0429 (13)	0.0386 (14)	0.0456 (14)	−0.0053 (11)	−0.0042 (12)	0.0092 (12)
C9	0.0406 (15)	0.0624 (19)	0.0651 (17)	−0.0106 (15)	−0.0001 (14)	0.0153 (16)
C10	0.0554 (18)	0.0652 (19)	0.0655 (19)	0.0109 (16)	0.0184 (15)	0.0144 (16)
C11	0.075 (2)	0.0508 (17)	0.0405 (15)	0.0094 (15)	0.0100 (14)	0.0007 (13)
C12	0.0569 (16)	0.0379 (14)	0.0353 (13)	−0.0015 (13)	−0.0050 (12)	0.0054 (11)
C13	0.0647 (18)	0.0464 (17)	0.0607 (17)	−0.0199 (14)	−0.0066 (15)	−0.0008 (14)
C14	0.080 (2)	0.0604 (19)	0.0521 (16)	−0.0142 (18)	−0.0114 (15)	−0.0103 (16)

### Geometric parameters (Å, °)

**Table:**

Cl1—C4	1.731 (3)	C7—C8	1.393 (3)
S1—O2	1.4228 (18)	C7—C12	1.401 (3)
S1—O1	1.4316 (17)	C8—C9	1.393 (4)
S1—N1	1.626 (2)	C8—C13	1.504 (3)
S1—C1	1.764 (2)	C9—C10	1.375 (4)
N1—C7	1.440 (3)	C9—H9	0.9300
N1—H1N	0.818 (16)	C10—C11	1.356 (4)
C1—C2	1.383 (3)	C10—H10	0.9300
C1—C6	1.385 (3)	C11—C12	1.383 (4)
C2—C3	1.380 (4)	C11—H11	0.9300
C2—H2	0.9300	C12—C14	1.498 (4)
C3—C4	1.364 (4)	C13—H13A	0.9600
C3—H3	0.9300	C13—H13B	0.9600
C4—C5	1.378 (4)	C13—H13C	0.9600
C5—C6	1.375 (3)	C14—H14A	0.9600
C5—H5	0.9300	C14—H14B	0.9600
C6—H6	0.9300	C14—H14C	0.9600
			
O2—S1—O1	120.37 (12)	C12—C7—N1	118.2 (2)
O2—S1—N1	106.12 (12)	C7—C8—C9	117.5 (2)
O1—S1—N1	106.90 (10)	C7—C8—C13	122.8 (2)
O2—S1—C1	107.66 (12)	C9—C8—C13	119.7 (2)
O1—S1—C1	107.05 (11)	C10—C9—C8	120.8 (3)
N1—S1—C1	108.29 (11)	C10—C9—H9	119.6
C7—N1—S1	121.78 (16)	C8—C9—H9	119.6
C7—N1—H1N	113.2 (19)	C11—C10—C9	120.7 (3)
S1—N1—H1N	115.5 (19)	C11—C10—H10	119.7
C2—C1—C6	120.6 (2)	C9—C10—H10	119.7
C2—C1—S1	120.11 (19)	C10—C11—C12	121.4 (3)
C6—C1—S1	119.31 (19)	C10—C11—H11	119.3
C3—C2—C1	119.3 (3)	C12—C11—H11	119.3
C3—C2—H2	120.4	C11—C12—C7	117.7 (2)
C1—C2—H2	120.4	C11—C12—C14	119.1 (2)
C4—C3—C2	119.8 (3)	C7—C12—C14	123.1 (2)
C4—C3—H3	120.1	C8—C13—H13A	109.5
C2—C3—H3	120.1	C8—C13—H13B	109.5
C3—C4—C5	121.3 (3)	H13A—C13—H13B	109.5
C3—C4—Cl1	119.4 (2)	C8—C13—H13C	109.5
C5—C4—Cl1	119.3 (2)	H13A—C13—H13C	109.5
C6—C5—C4	119.4 (3)	H13B—C13—H13C	109.5
C6—C5—H5	120.3	C12—C14—H14A	109.5
C4—C5—H5	120.3	C12—C14—H14B	109.5
C5—C6—C1	119.6 (2)	H14A—C14—H14B	109.5
C5—C6—H6	120.2	C12—C14—H14C	109.5
C1—C6—H6	120.2	H14A—C14—H14C	109.5
C8—C7—C12	121.8 (2)	H14B—C14—H14C	109.5
C8—C7—N1	120.0 (2)		
			
O2—S1—N1—C7	174.71 (18)	S1—C1—C6—C5	−175.2 (2)
O1—S1—N1—C7	45.1 (2)	S1—N1—C7—C8	97.6 (2)
C1—S1—N1—C7	−69.9 (2)	S1—N1—C7—C12	−83.4 (3)
O2—S1—C1—C2	9.0 (2)	C12—C7—C8—C9	3.2 (4)
O1—S1—C1—C2	139.75 (19)	N1—C7—C8—C9	−177.8 (2)
N1—S1—C1—C2	−105.3 (2)	C12—C7—C8—C13	−175.3 (2)
O2—S1—C1—C6	−172.5 (2)	N1—C7—C8—C13	3.7 (4)
O1—S1—C1—C6	−41.8 (2)	C7—C8—C9—C10	−1.1 (4)
N1—S1—C1—C6	73.1 (2)	C13—C8—C9—C10	177.4 (2)
C6—C1—C2—C3	−2.8 (4)	C8—C9—C10—C11	−1.2 (4)
S1—C1—C2—C3	175.7 (2)	C9—C10—C11—C12	1.5 (4)
C1—C2—C3—C4	0.2 (4)	C10—C11—C12—C7	0.5 (4)
C2—C3—C4—C5	2.0 (4)	C10—C11—C12—C14	−178.4 (3)
C2—C3—C4—Cl1	−177.7 (2)	C8—C7—C12—C11	−2.9 (4)
C3—C4—C5—C6	−1.5 (4)	N1—C7—C12—C11	178.1 (2)
Cl1—C4—C5—C6	178.1 (2)	C8—C7—C12—C14	175.9 (2)
C4—C5—C6—C1	−1.1 (4)	N1—C7—C12—C14	−3.1 (4)
C2—C1—C6—C5	3.3 (4)		

### Hydrogen-bond geometry (Å, °)

**Table:**

*D*—H···*A*	*D*—H	H···*A*	*D*···*A*	*D*—H···*A*
N1—H1N···O1^i^	0.82 (2)	2.28 (2)	3.083 (3)	166 (3)

Symmetry codes: (i) −*x*+2, *y*−1/2, −*z*+1/2.
